# Cuproptosis-Related Genes Are Associated with Cell Cycle and Serve as the Prognostic Signature for Clear Cell Renal Cell Carcinoma

**DOI:** 10.3390/jcm11247507

**Published:** 2022-12-18

**Authors:** Tuanjie Guo, Jian Zhang, Zhihao Yuan, Heting Tang, Tao Wang, Xiang Wang, Siteng Chen

**Affiliations:** 1 Department of Urology, Shanghai General Hospital, Shanghai Jiao Tong University School of Medicine, Shanghai 200080, China; 2 Department of Urology, Renji Hospital, Shanghai Jiao Tong University School of Medicine, Shanghai 200080, China

**Keywords:** cuproptosis, clear cell renal cell carcinoma, prognosis, signature, cell cycle

## Abstract

Cuproptosis is a newly discovered type of cell death. The role and potential mechanism of Cuproptosis-related genes (CRGs) in the prognosis of cancer patients are not fully understood. In this study, we included two cohorts of clear cell renal cell carcinoma patients, TCGA and E-MTAB-1980. The TCGA cohort is used as a training set to construct a CRG signature using the LASSO-cox regression analysis, and E-MTAB-1980 is used as a cohort for verification. A total of eight genes (*FDX1*, *LIAS*, *LIPT1*, *DLAT*, *PDHA1*, *MTF1*, *GLS*, *CDKN2A*) were screened to construct a prognostic model in the TCGA cohort. There is a significant difference in OS (*p* < 0.0001) between the high and low cuproptosis score group, and a similar difference is also observed in the OS (*p* = 0.0054) of the E-MTAB-1980 cohort. The area under the ROC curves (AUC) were 0.87, 0.82, and 0.78 at 1, 3, and 5 years in the TCGA cohort, respectively. Finally, gene set enrichment analysis revealed that CRGs were associated with cell cycle and mitotic signaling pathways.

## 1. Introduction

Renal carcinoma is a solid tumor with an increasing incidence year by year. According to US statistics, it is estimated that there will be 76,080 new cases and 13,780 deaths in 2022 [1]. Renal cell carcinoma (RCC) is the most common form of renal carcinoma, accounting for 85% of cases. It is more common in men than women (1.7:1), and most people are older, with an average age of 64 [2]. The most common subtype of renal cell carcinoma is clear cell renal cell carcinoma (ccRCC), which is a highly aggressive tumor [3]. Although patients with early detection and surgical resection have a better prognosis, many patients have progressed when diagnosed as tumors due to atypical symptoms of RCC [4]. The markers currently used to evaluate the prognosis of patients with ccRCC mainly include the protein staining of the proliferation markers Ki-67, p53, and PTEN, as well as the hypoxia-inducible factor pathway factors carbonic anhydrase IX and the VEGF family. In addition, somatic mutation, gene methylation, gene expression data, germ cell variation, and immune biomarkers such as CD8 and PD-L1 are also associated with prognosis [5]. However, none have been widely verified and applied. In addition, there is no effective model to evaluate the prognosis of patients. Therefore, the construction of a clinical survival prognosis model for ccRCC patients has an extremely important role in evaluating the prognosis of patients.

Recently, Tsvetkov et al. found that copper-dependent cell death is a new type of cell death that is different from the known cell death mechanism. This copper-dependent cell death occurs through the direct binding of copper ions to fatty acylated components of the tricarboxylic acid cycle (TCA) of mitochondrial respiration. The binding of copper ions leads to the accumulation of fatty acylated proteins and subsequent downregulation of iron-sulfur cluster proteins. This process further leads to proteotoxic stress, which ultimately leads to cell death [6].

The discovery of a new cell death pathway is of great significance, which means that cancer can be treated by inducing cancer cells to die in this way. Therefore, it is necessary to elaborate on the relationship between the cell death pathway and the prognosis of cancer patients. As cuproptosis is a newly discovered cell death, the relationship between cuproptosis and cancer is still unclear. Whether CRGs can construct a prognostic model for cancer patients is also a meaningful research direction.

In this study, we constructed a prognostic model using CRGs. We found that the model can effectively predict the prognosis of patients. Furthermore, we explored the mechanism of the prognostic model and found that the signaling pathways associated with CRGs were the cell cycle and mitosis.

## 2. Materials and Methods

### 2.1. Data Sources

**Figure 1:**
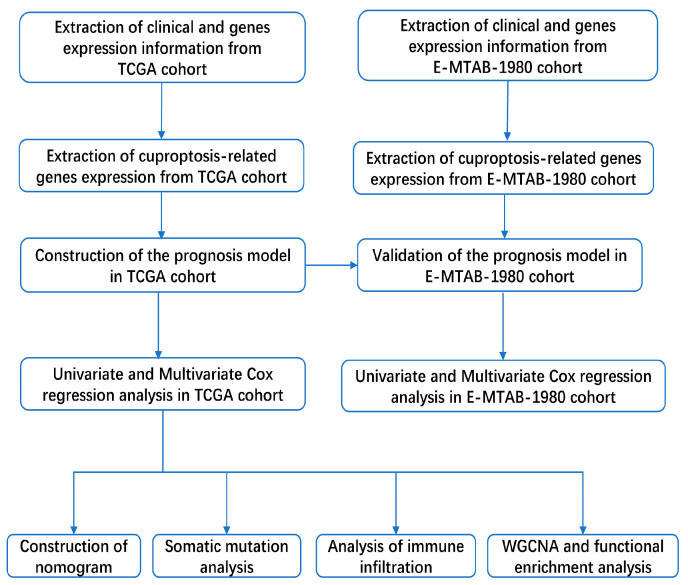
The workflow figure of this study.

In this study, a total of two cohorts of patients with ccRCC were included; they are The Cancer Genome Atlas (TCGA, https://portal.gdc.cancer.gov/, accessed on 28 March 2022) and E-MTAB-1980 (https://www.ebi.ac.uk/, accessed on 28 March 2022) [7]. Only when the patient’s gene expression information and clinical information are complete will the patient be included in the study. A total of 531 patients with ccRCC were included in the TCGA cohort, and their mRNA sequencing data and the corresponding clinical information were downloaded from the TCGA database. In the E-MTAB-1980 cohort, a total of 101 ccRCC patients’ mRNA sequencing data and corresponding clinical information were extracted from the E-MTAB-1980 database. The clinical information of the two cohorts was described in the Supplementary Material S1. Simultaneously, ten CRGs were extracted from the findings of Tsvetkov et al. [6], and all genes could be found in the Supplementary Material S2. Our workflow is shown in Figure 1.

### 2.2. Development of the Prognosis Model Based on CRGs

Extracting ten CRGs from the TCGA cohort, we used the *glmnet* package to perform a least absolute contraction and selection operator (LASSO) Cox regression analysis in the R environment to find specific CRGs significantly related to the clinical prognosis of patients. The coefficient of each gene was consistent with the weight calculated according to the prognostic model. We set the lambda to 1000 when calculating the coefficients to ensure the robustness of the model. The formula for the cuproptosis score was as follows:

$$Cuprotosis\;score=\sum_{i=1}^{n}\left(Coef*Cup\right)\;$$

The Coef represented the weight of each prognosis-associated cuproptosis gene, and Cup represented the mRNA expression of these genes. According to the median of the cuproptosis score, patients were divided into a high-risk group and a low-risk group. Subsequently, we validated this survival prognostic model in the E-MTAB-1980 cohort with the same method.

### 2.3. Univariate and Multivariate Cox Regression Analysis and Survival Analysis

Univariate and multivariate Cox regression were implemented through the *dplyr* and *survival* packages. The survival analysis was implemented through *survival* and *survminer* packages.

### 2.4. Construction of Nomogram Prognostic Model

To conveniently and intuitively predict patient outcomes, we constructed a nomogram prediction model based on the method described in our previous study [8,9].

### 2.5. Somatic Mutation Analysis

Mutation data from patients with ccRCC were extracted from the TCGA database (https://portal.gdc.cancer.gov/), and an analysis of the top 20 mutated genes for differences between different groups was performed in the R environment using the *maftools* package.

### 2.6. Immune Cell Infiltration

The CIBERSORT algorithm was used to evaluate the immune infiltration of patients with ccRCC. According to the instructions [10], the LM22 file was used as the signature of immune cells, and the permutations were set to 1000.

### 2.7. Functional Enrichment Analysis

To explore the possible mechanism related to the cuproptosis score and prognosis, we used a weighted gene co-expression network analysis (WGCNA) based on differentially expressed genes (|fold change| > 2, *p* < 0.05) in patients with ccRCC [8]. The correlation between gene modules and clinicopathological features was calculated to identify the module most relevant to the cuproptosis score. Finally, through the Kyoto Encyclopedia of Genes and Genomes (KEGG) and genetic ontology (GO) analysis, we explored the potential biological mechanism of CRGs that might participate in Metascape [11]. By uploading differentially expressed genes, Metascape enabled automated gene set enrichment analysis.

### 2.8. Statistical Analysis

In this study, we used the Mann–Whitney U test to analyze the difference between continuous variables and the ANOVA test for continuous variables of more than two groups. Kaplan–Meier curve analysis was used to compare OS with the log-rank test. The Spearman test was used to test for correlation. All statistical analyses were performed in R (4.0.0) and GraphPad Prism 8. All statistical *p* values were two-sided *p* < 0.05, indicating statistical significance.

## 3. Results

### 3.1. Construction and Verification of the Prognostic Model Based on CRGs

**Figure 2:**
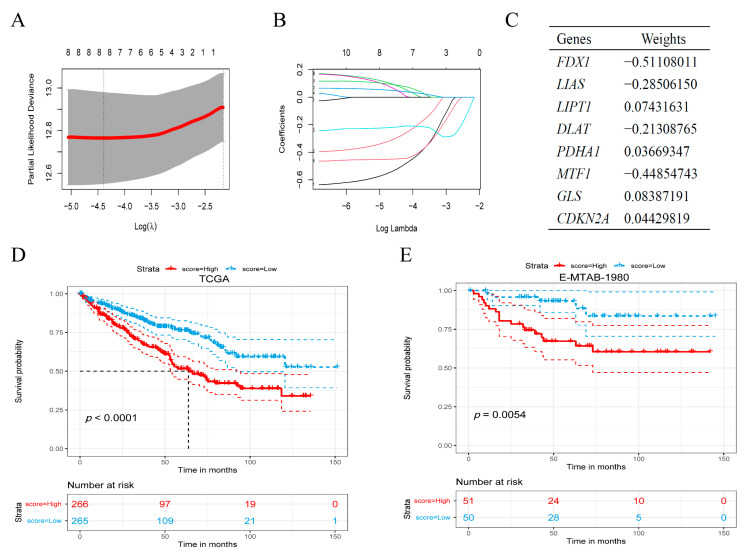
Establishment of prognostic model. (**A**,**B**) The tenfold cross-validated error and coefficients at varying levels of penalization plotted against the log (lambda) sequence for the least absolute shrinkage and selection operator analysis. (**C**) Selected genes and associated weights in the prognosis model. (**D**) Kaplan–Meier survival analysis of overall survival in the TCGA cohort. (**E**) Kaplan–Meier survival analysis to validate the prognostic model in the E-MTAB-1980 cohort.

We used the survival information and the expression of CRGs of the patients in the TCGA cohort to screen prognostic-related genes based on LASSO-cox regression analysis (Figure 2A,B). Finally, we screened eight genes to construct a prognostic model, and these eight genes and their related weights are shown in Figure 2C. Then we divided the patients into high-score and low-score groups according to the median of the cuproptosis score in the TCGA cohort. There was a significant difference in OS (*p* < 0.001) between the high-score group and the low-score group (Figure 2D). Then, we validated this model in the E-MTAB-1980 cohort (Figure 2E), and the result showed that the OS of different score groups also had a strongly significant difference (*p* = 0.0054).

### 3.2. Cuproptosis Score Correlates with Disease Progression

**Figure 3:**
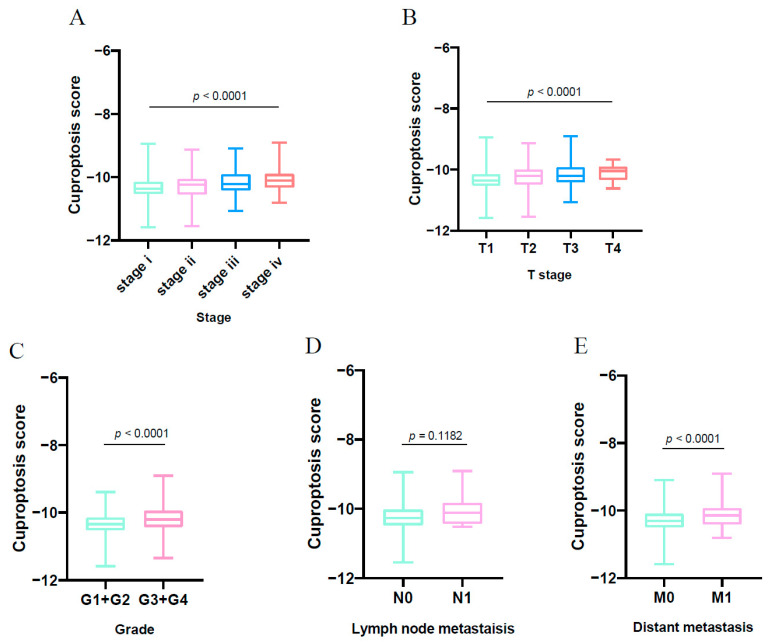
Relationship between cuproptosis score and different clinicopathological information groups based on TCGA cohort. (**A**) The relationship between cuproptosis score and stage. (**B**) The relationship between cuproptosis score and T stage. (**C**) The relationship between cuproptosis score and grade. (**D**) The relationship between cuproptosis score and lymph node metastasis. (**E**) The relationship between cuproptosis score and distant metastasis.

Based on the clinical data of ccRCC patients in the TCGA cohort, we analyzed the relationship between a patient’s T stage, grade, stage, lymph node metastasis, and distant metastasis and cuproptosis score. We found that the stage and the T stage of the patients gradually increased with increasing cuproptosis score (Figure 3A,B). Furthermore, we also found that with increasing cuproptosis score, the grade of the patients also gradually increased (Figure 3C). We also found that the cuproptosis score of the patients was related to distant metastasis but not lymph node metastasis, which might be caused by fewer patients with lymph node metastasis (Figure 3D,E).

### 3.3. Cuproptosis Score Can Be Used as an Independent Prognostic Factor

**Figure 4:**
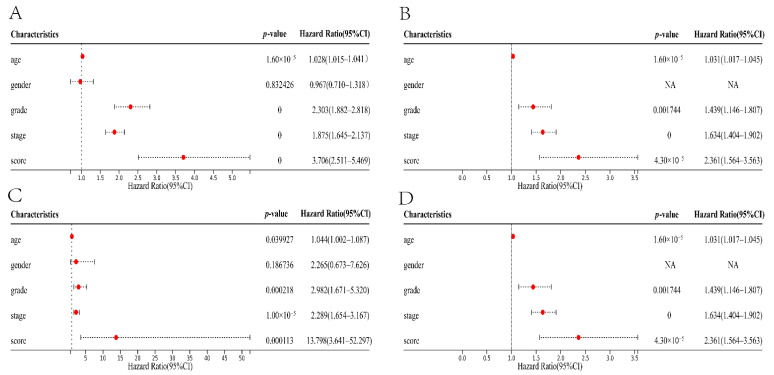
Univariate and multivariate Cox regression analysis. (**A**,**B**) Univariate and multivariate Cox regression analysis of the TCGA cohort. (**C**,**D**) Univariate and multivariate Cox regression analysis of the E-MTAB-1980 cohort.

In two independent cohorts of ccRCC patients, we used univariate and multivariate Cox regression analysis to assess whether the cuproptosis score could be used as an independent prognostic factor. We found that in the TCGA cohort, whether it is univariate (hazard ratio = 3.706, 95% CI 2.511–5.469, *p* < 0.0001) or multivariate (hazard ratio = 2.361, 95% CI 1.564–3.563, *p* < 0.0001) Cox regression analysis, the cuproptosis score had important significance in OS of patients (Figure 4A,B). Homoplastically, we also obtained the same result in the E-MTAB-1980 cohort (Figure 4C,D).

### 3.4. Construction and Evaluation of Nomogram Based on Cuproptosis Score

**Figure 5:**
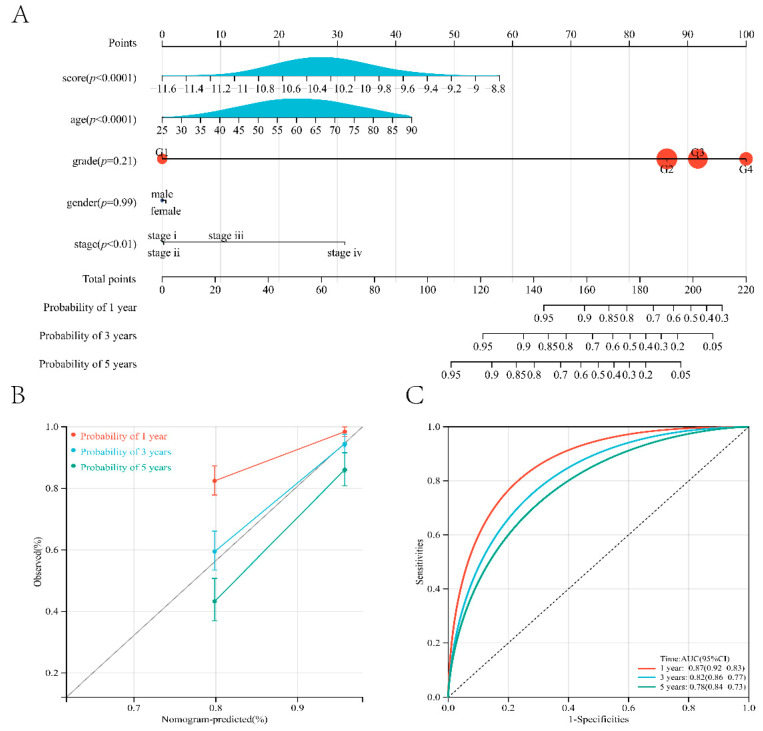
Nomogram prediction model predicts the prognosis of patients. (**A**,**B**) Nomogram based on the prognostic model and clinicopathological information. (**C**) ROC curve of the TCGA cohort.

To apply our prognostic model to predict the survival time of ccRCC patients, we constructed a nomogram prognostic model (Figure 5A). Through the nomogram prognostic model, we could intuitively predict the survival probability of patients for 1, 3, and 5 years. The nomogram model we constructed also achieved high efficiency, and the accuracy rates of predicting the survival time of patients at 1, 3, and 5 years were 0.87, 0.82, and 0.78, respectively.

### 3.5. Revealing Differences in Somatic Mutation between Different Cuproptosis Scores

**Figure 6:**
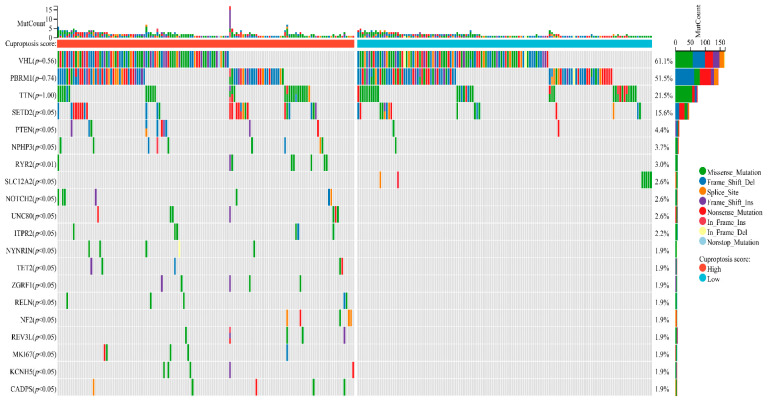
Analysis of gene mutation differences between different cuproptosis score groups.

To explore differences in somatic mutations between different cuproptosis scores, we performed mutational analysis on the top twenty genes with the highest mutation frequency in ccRCC tumors. We found that the top three genes with the highest mutation frequency (*VHL*, *PBRM1*, and *TTN*) did not differ between the two groups. The remaining 17 genes with higher mutation frequencies had statistically significant mutation differences between the two groups (Figure 6). In addition to this, mutations were more likely to occur in the group with a higher cuproptosis score.

### 3.6. Cuproptosis Score Correlates with Immune Cell Infiltration

**Figure 7:**
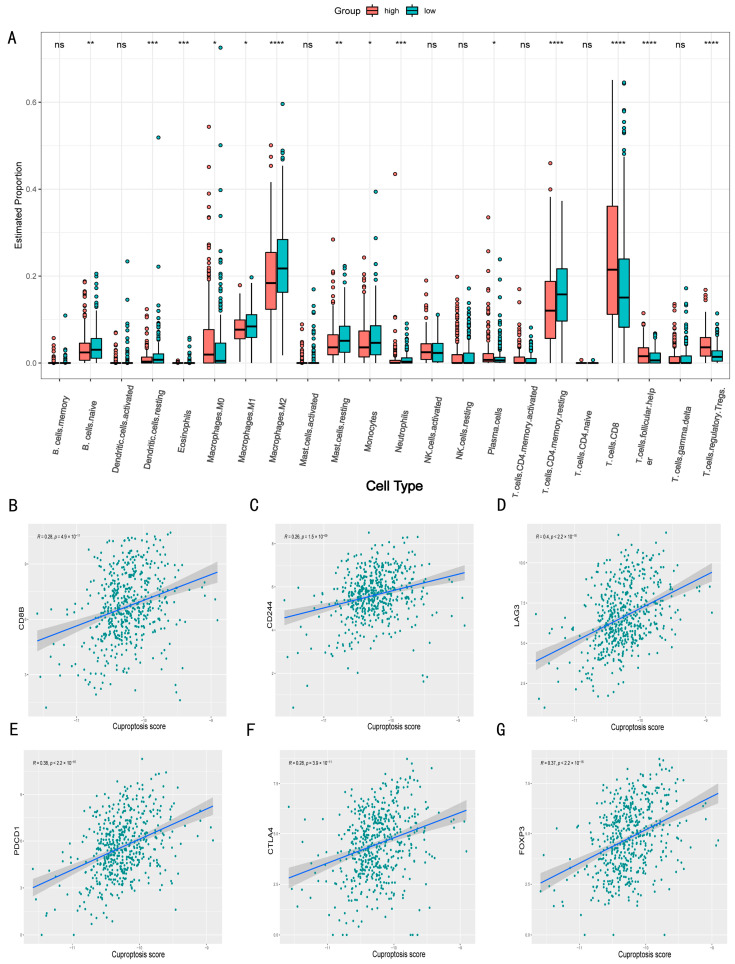
Analysis of immune cell infiltration. (**A**) The difference in the proportion of immune cells between the high-risk group and the low-risk group. (**B**–**G**) Correlation analysis of immune cell markers and immune checkpoints with cuproptosis score. *: *p* < 0.05; **: *p* < 0.01; ***: *p* < 0.001; ****: *p* < 0.0001; ns: no significance.

To evaluate whether the cuproptosis score was related to different immune cell infiltration, we evaluated the proportion of immune cells in the tumor microenvironment of patients with different cuproptosis scores (Figure 7A). We found that compared to the low cuproptosis score group, the high cuproptosis score group had more CD8^+^ T cells and Treg cells, lower CD4^+^ T memory cells, M2 macrophages, and dendritic cells. This could be related to Treg cells that regulated cytotoxic T cell exhaustion [12,13]. To validate our analysis, we performed a correlation analysis of immune cell surface markers and immune checkpoints with the cuproptosis score and found that the expression of the CD8B and T cell exhaustion markers CD244, LAG3, and PDCD1 were significantly positively correlated, which was consistent with the result that the high-score group had more CD8^+^ T cell infiltration (Figure 7B–E). Furthermore, the expression of the Treg cell markers FOXP3 and CTLA4 was also strongly correlated with the cuproptosis score, which also verified that CRGs could inhibit the killing function of cytotoxic T cells by regulating Treg cells (Figure 7F,G).

### 3.7. WGCNA Reveals That CRGs Are Related to Cell Cycle and Mitosis

**Figure 8:**
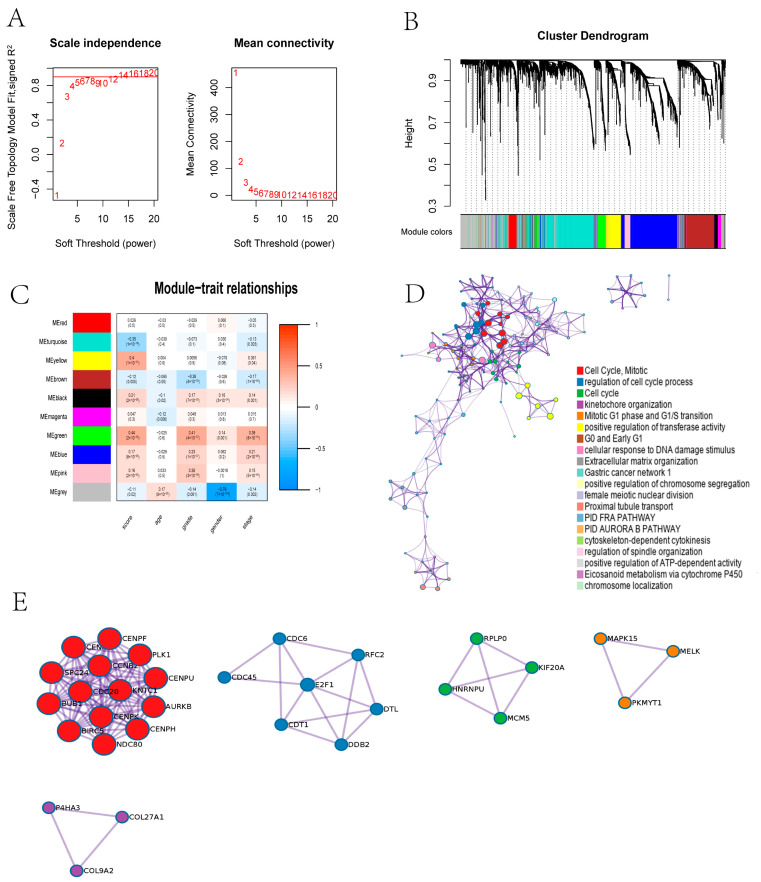
Gene weight co-expression network analysis and gene function enrichment analysis. (**A**) Soft power estimation in ccRCC for WGCNA. (**B**) Gene dendrogram with different colors showing the modules identified by WGCNA. (**C**) The relationship between gene modules and clinical characteristics. (**D**) Potentially enriched pathways of the co-expressed genes in green and yellow modules. (**E**) The protein–protein interaction network of the top five signaling pathways.

Finally, based on WGCNA, we conducted a functional enrichment analysis of differentially expressed genes in ccRCC patients (Figure 8A). Subsequently, we modularized the enriched genes (Figure 8B). By associating the modular genes with the clinical characteristics and cuproptosis score, we found that the green and yellow modules had the highest correlation with the cuproptosis score (Figure 8C). Finally, we analyzed the genes of the green and yellow modules through GO and KEGG and found that the enriched genes were related to the cell cycle and mitosis signaling pathways (Figure 8D). The protein–protein interaction networks related to the top five signaling pathways are also shown (Figure 8E).

## 4. Discussion

As we know, ccRCC is a highly aggressive tumor. At present, apart from the traditional TNM stage and Fuhrman grade system that could be used as a reference factor for the patient’s prognosis [14], there is no other prognostic factor that can predict the survival time of the patient. Therefore, it is urgent to develop a tool that can predict the prognosis of ccRCC patients. The use of CRGs to construct a prognostic model in ccRCC has been reported, but the previous studies lacked validation by other cohorts [15,16,17]. In addition to that, they did not consider CRGs as a whole to explore the potential biological mechanism for predicting the prognosis.

We included eight cuproptosis-related genes (*FDX1*, *LIAS*, *LIPT1*, *DLAT*, *PDHA1*, *MTF1*, *GLS*, *CDKN2A*) to construct a prognostic model. Among them, *CDKN2A* mutation played an important role in ccRCC metastasis by affecting the expression of p16/p14, and the phosphorylation of PDHA1 was related to disease progression [18,19]. The mechanism between the remaining six genes and the occurrence and progression of renal cell carcinoma has not been explored. Particularly, *FDX1* and *MTF1* with larger weights and their relationship with ccRCC deserved further study.

Our prognostic model based on eight CRGs could well classify patients with high and low risks, which was verified in both public databases. Therefore, it had high credibility. Furthermore, we found that the cuproptosis score was associated with disease progression in patients with ccRCC. Multivariate Cox analysis also revealed that the cuproptosis score could be used as an independent prognostic factor in patients with ccRCC. The nomogram prediction model could also accurately predict the survival time of the patients, which had potential application value for the clinical treatment of patients with ccRCC.

Because of the dual roles that copper ion present, cells can only survive in a certain range of concentration. The mechanism by which excessive copper ions led to cell death had been a mystery. Tsvetkov et al. found that copper ions that reach mitochondria through copper carriers directly bind to fatty acylated proteins, causing them to form long chains and agglomerates, leading to cell death. These copper ions also interfered with sulfide clusters, resulting in the down-regulation of iron sulfide proteins, leading to cytotoxicity stress and death [6]. This provided a new direction for the treatment of cancer patients by inducing copper overload in cancer cells and leading to cuproptosis, thus achieving the purpose of killing cancer cells. We revealed that CRGs could construct prognostic models for cancer patients. This also provided a reference for the study of cuproptosis and cancer.

To date, the relationship between CRGs and the exhaustion of the immune microenvironment has not been explored. We found that the tumor microenvironment of patients with a higher cuproptosis score tended to show an immunosuppressive T cell exhaustion phenotype. Therefore, this discovery provides new insights into the mechanisms of T cell exhaustion. In the enrichment analysis, we used WGCNA to study CRGs as a whole and found that CRGs were related to cell cycle and mitosis signaling pathways. This finding also deserves further exploration.

The application value of this prognostic model was reflected mainly in medical institutions that can detect the expression of CRGs in tumor samples of patients with ccRCC, insert them into the prognosis formula, and then perform a personalized prognosis evaluation of patients with ccRCC by nomogram.

Although our constructed model had important significance for the prognosis of ccRCC patients, our research inevitably had some shortcomings. For example, the cohorts we used were all shared from public databases, which would inevitably cause some deviation. The prognostic model constructed in this study was based on RNA sequencing data from public data, and the lack of validation of the expression levels of the screening genes in clinical samples is also a notable limitation. Additionally, our analysis of potential mechanisms still needs further experimental research.

## 5. Conclusions

We used the CRGs to establish a survival prognosis model for ccRCC patients, which could effectively predict the prognosis of patients. Mechanically, through immune infiltration analysis and functional enrichment analysis, we found that CRGs could control tumor progression by regulating T cell exhaustion and cell cycle.

## Data Availability

In this study, we included two public datasets, which are the E-MTAB-1980 (https://www.ebi.ac.uk/) and The Cancer Genome Atlas (TCGA, https://portal.gdc.cancer.gov/). The datasets used and analyzed in this study are also available from the corresponding author upon reasonable request.

## Supplementary Materials

The following supporting information can be downloaded at: https://www.mdpi.com/article/10.3390/jcm11247507/s1, S1: Basic clinical characteristics of patients in the TCGA cohort and the E-MTAB-1980 cohort. S2: Cuproptosis-related genes.
