# Supplementary material for: Cuproptosis-Related Genes Are Associated with Cell Cycle and Serve as the Prognostic Signature for Clear Cell Renal Cell Carcinoma
Source: J Clin Med. 2022 Dec 18;11(24):7507. doi: 10.3390/jcm11247507 (PMC9782129; doi:10.3390/jcm11247507)
Supplement: Supplementary file 1 [file jcm-11-07507-s001.zip › jcm-2070119-supplementary.pdf]

Supplementary Material

S1: Basic clinical characteristics of patients in the TCGA cohort and E-MTAB-1980 cohort.

	TCGA (531)	E-MTAB-1980 (101)
Age(years)		
≥65	198(37.3%)	44(43.6%)
< 65	333(62.7%)	57(56.4%)
Sex		
Male	345(65.0%)	77(23.8%)
Female	186(35.0%)	24(23.8%
Grade		
G1	13(2.4%)	13(12.9%)
G2	229(43.1%)	59(58.4%)
G3	205(38.6%)	22(21.8%)
G4	76(14.3%)	5(5.0%)
Unknown	8(1.5%)	2(2.0%)
Stage		
I	266(50.1%)	67(66.3%)
II	57(10.7%)	11(10.9%)
III	124(23.4%)	14(13.9%)
IV	84(15.8%)	13(12.9%)
T stage		
T1	271(51.0%)	68(67.3%)
T2	69(13.0%)	11(10.9%)
T3	180(33.9%)	21(20.85)
T4	11(2.1%)	1(1.0%)
N stage		
N2	/	4(4%)
N1	16(3.0%)	3(3.0%)
N0	240(45.2%)	94(93.1%)
Unknown	275(51.8%)	/
M stage		
M1	79(14.9%)	12(11.9%)
M0	422(79.5%)	89(88.1%)
Unknown	30(5.6%)	/
Survival		
Dead	175(33.0%)	23(22.8%)
Living	356(67.0%)	78(77.25)

S2: Cuproptosis related genes.

FDX1: ferredoxin 1, this gene encodes a small iron-sulfur protein that transfers electrons from NADPH through ferredoxin reductase to mitochondrial cytochrome P450, involved in steroid, vitamin D, and bile acid metabolism.

LIAS: lipoic acid synthetase, the protein encoded by this gene belongs to the biotin and lipoic acid synthetases family.

LIPT1: lipoyltransferase 1, the process of transferring lipoic acid to proteins is a two-step process. The first step is the activation of lipoic acid by lipoate-activating enzyme to form lipoyl-AMP. For the second step, the protein encoded by this gene transfers the lipoyl moiety to apoproteins.

DLD: dihydrolipoamide dehydrogenase, this gene encodes a member of the class-I pyridine nucleotide-disulfide oxidoreductase family. The encoded protein has been identified as a moonlighting protein based on its ability to perform mechanistically distinct functions.

DLAT: dihydrolipoamide S-acetyltransferase, this gene encodes component E2 of the multi-enzyme pyruvate dehydrogenase complex (PDC). PDC resides in the inner mitochondrial membrane and catalyzes the conversion of pyruvate to acetyl coenzyme A.

PDHA1: pyruvate dehydrogenase (lipoamide) alpha 1, the pyruvate dehydrogenase (PDH) complex is a nuclear-encoded mitochondrial multienzyme complex that catalyzes the overall conversion of pyruvate to acetyl-CoA and CO (2), and provides the primary link between glycolysis and the tricarboxylic acid (TCA) cycle.

PDHB: pyruvate dehydrogenase (lipoamide) beta, the pyruvate dehydrogenase (PDH) complex is a nuclear-encoded mitochondrial multienzyme complex that catalyzes the overall conversion of pyruvate to acetyl-CoA and carbon dioxide, and provides the primary link between glycolysis and the tricarboxylic acid (TCA) cycle.

MTF1: metal-regulatory transcription factor 1, this gene encodes a transcription factor that induces expression of metallothioneins and other genes involved in metal homeostasis in response to heavy metals such as cadmium, zinc, copper, and silver.

GLS: glutaminase, this gene encodes the K-type mitochondrial glutaminase. The encoded protein is a phosphate-activated amidohydrolase that catalyzes the hydrolysis of glutamine to glutamate and ammonia.

CDKN2A: cyclin-dependent kinase inhibitor 2A, product encoded by this gene, through the regulatory roles of CDK4 and p53 in cell cycle G1 progression, share a common functionality in cell cycle G1 control.
